# Does smoking affect the prevalence of caffeine use in schizophrenia?

**DOI:** 10.1192/j.eurpsy.2022.2006

**Published:** 2022-09-01

**Authors:** H. Becerra Darriba

**Affiliations:** Osasunbidea - Servicio Navarro de Salud, Psychiatry - Centro De Salud Mental De Tudela, Tudela, Spain

**Keywords:** caffeine, tobacco, schizophrénia, Xanthine

## Abstract

**Introduction:**

Caffeine acts as a competing antagonist of adenosine receptors, increasing the release of norepinephrine and the activation of noradrenergic neurons. Long-standing schizophrenia patients frequently develop a comorbidly high daily caffeine intake. This could be explained by its relationship with smoking [1,2].

**Objectives:**

To determine caffeine consumption in schizophrenia and predisposing factors.

**Methods:**

Cross-sectional study designed on a sample of 68 outpatients with a follow-up of at least 5 years at the Mental Health Unit, aged between 18 and 65 years, diagnosed with schizophrenia (ICD-10). Average daily caffeine intake was quantified by reference values for each beverage: coffee (66.7mg/100ml), tea (30mg/100ml), soft or energy drinks (11.5mg/100ml). High intake was defined as a consumption of ≥200mg of caffeine per day. Retrospective review of medical records revealed tobacco use and negative symptoms observed on the PANSS scale. Statistical analysis were performed using SPSS v21.0 (significance p<0.05).

**Results:**

88.2% of the subjects were daily caffeine consumers with a mean intake of 146.7mg/day (SD=5.8), and a mean consumption time of 6.2 years. Coffee was the predominant beverage in 66.7% of the cases, followed by soft or energy drinks (25%) and tea (0.1%). 45% of participants also had a high caffeine intake of ≥200 mg/day. Comorbid smoking was found in 93% of these patients. Negative symptomatology prevailed among caffeine consumers (PANSS-N= 41.3).

**Conclusions:**

Xanthine abuse seems to be highly prevalent in people with schizophrenia, and there may be a relationship with smoking and negative psychotic symptoms.

**Disclosure:**

No significant relationships.

